# Coexistence of Eutopic Thyroid Gland and Ectopic Thyroid Tissue: Rare but Not Uncommon

**DOI:** 10.7759/cureus.59834

**Published:** 2024-05-07

**Authors:** Badriya Al Suqri, Asya Al Busaidi, Ahmed Al Dhuhli

**Affiliations:** 1 Nuclear Medicine, Royal Hospital, Muscat, OMN; 2 Radiology, Sultan Qaboos Comprehensive Cancer Care and Research Centre, Muscat, OMN; 3 Radiology, Royal Hospital, Muscat, OMN

**Keywords:** ultrasound, spect-ct, thyroid pathologies, thyroid scan, ectopic thyroid tissue

## Abstract

Ectopic thyroid tissue is very rare, but the coexistence of ectopic and eutopic thyroid glands is even more rare. The recognition of this diagnosis is important in patients who are being treated for thyrotoxicosis, but it is also crucial to exclude other associated serious disease conditions.

In this article, we report three different cases that showed ectopic thyroid tissue with the coexisting presence of an eutopic thyroid gland. All three cases showed different outcomes. The recognition of this condition is of great importance because it alerts the referring physicians to this rare, yet possible occurrence and the potential pathological conditions associated with it.

The first case showed how imaging could help outline even small ectopic thyroid tissue outside of the neck region even in cases where histopathological confirmation is difficult. The second case was very rare as thyroid carcinoma originated in ectopic thyroid tissue. In the last case, the initial imaging was misleading as it mimicked greatly ectopic thyroid tissue, and only detailed history and careful inspection of the images could lead to the correct interpretation of the findings.

## Introduction

Ectopic thyroid glands are a rare entity. They may be the only functioning gland; however, they may sometimes coexist with an eutopic thyroid gland [1]. We report three cases of incidentally discovered simultaneous presence of a normally positioned thyroid gland and ectopic thyroid tissue on thyroid uptake scintigraphy.

Two reported cases were referred for thyroid uptake scans as a workup for thyrotoxicosis. The remaining patient was referred for the evaluation of a neck lump.

## Case presentation

Case 1

Case History/Examination

A 50-year-old male patient with no significant past medical history presented with a three-month history of palpitations and shortness of breath. General examination revealed diffuse goiter and fine tremor.

Methods

ECG showed sinus tachycardia. Initial thyroid function test revealed free T4 of >100 pmol/L (normal: 8.4-22.6), very low TSH (<0.01), and positive thyroid peroxidase antibodies.

Imaging

**Figure 1:**
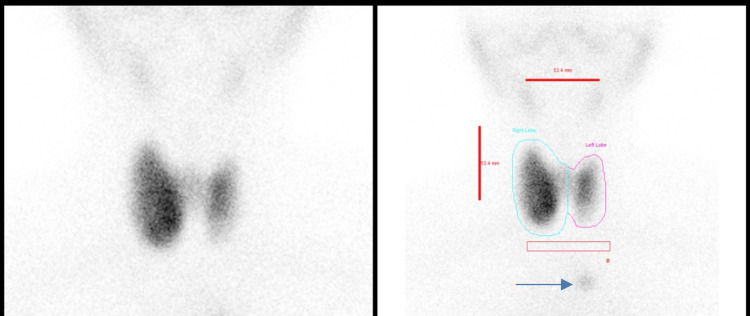
Planar images of thyroid uptake scintigraphy using TC99m pertechnetate showing thyroid gland uptake and an abnormal focal uptake in the upper part of the chest (blue arrow).

**Figure 2:**
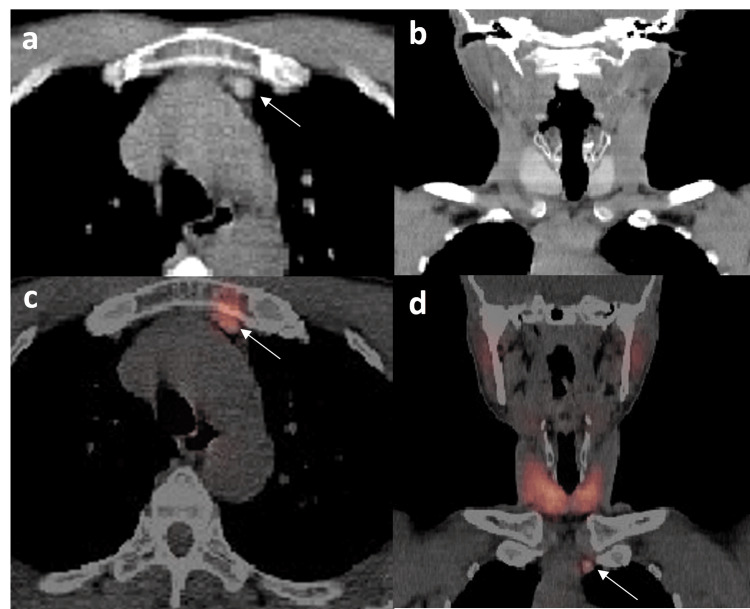
Axial CT (a), coronal CT (b), axial fused SPECT/CT (c), and coronal fused SPECT/CT, and (d) reconstruction of SPECT/CT scan of the neck and upper chest. Soft tissue nodule (white arrow) in the pre-vascular space with similar density and uptake to the thyroid gland.

Ultrasound of the neck was performed and it revealed diffuse goiter with a heterogenous pattern suggestive of thyroiditis associated with reactive upper cervical lymphadenopathy. The initial impression was thyrotoxicosis, and the patient was started on a regular dose of antithyroid medication with a good response. The patient was then referred for a thyroid uptake scan in which planar images demonstrated a bulky heterogenous right thyroid lobe with slightly increased uptake (global uptake of 4.86%, normal range: 0.4-4%), which could represent toxic goiter or hot nodule (Figure 1). An incidental interesting finding was noted in the planar images where a focal uptake was seen well below the left thyroid lobe. SPECT-CT (Figure 2) was performed to localize the focal uptake, and interestingly, it was seen to correspond to a small hyperdense soft tissue nodule measuring 0.7x0.7 cm in the left perivascular space. It showed similar density and uptake to the thyroid gland; hence, it is likely to represent ectopic thyroid tissue.

Conclusion and Results

Due to the nodule’s small size and pre-vascular location, it was difficult for sampling and histopathological confirmation. However, the imaging characteristics (density, position, and uptake) were sufficient to conclude the diagnosis of ectopic thyroid tissue. The patient was started on antithyroid medications for thyrotoxicosis and did well on them.

Case 2

Case History/Examination

A 39-year-old lady presented with painful swelling on the right side of the neck. On further inquiry, she also reported increased appetite and mood swings lately with no significant changes in bowel habits. Clinical examination revealed right supraclavicular firm swelling and unremarkable thyroid gland examination.

Methods

The thyroid function test was unremarkable. An ultrasound examination of the neck was done, and it showed a right thyroid lobe hypoechoic nodule measuring 1.8x1.4x1.2 cm. The nodule is solid and ill-defined with internal vascularity. Multifocal punctate calcifications were seen. Enlarged lymph nodes were noted in the right cervical region at levels III, IV, and V. The largest was seen in level V, supra-clavicular region, and measured 3.1x1.9 cm. It showed a heterogenous echo pattern with internal cystic changes and internal vascularity. The nodule was classified as TIRADS-5 with ipsilateral pathological lymphadenopathy.

Urgent fine needle aspiration cytology (FNAC) was obtained from the thyroid nodule and the right supraclavicular mass in the clinic. The result of the initial FNAC from the right thyroid nodule showed scanty benign thyroid follicles in a hemorrhagic background. The result of the sampling from the supraclavicular lesion came as normal thyroid tissue; hence, the possibility of ectopic thyroid tissue came into the differential diagnosis. Therefore, thyroid uptake scintigraphy was requested to confirm the presence of ectopic thyroid tissue.

**Figure 3:**
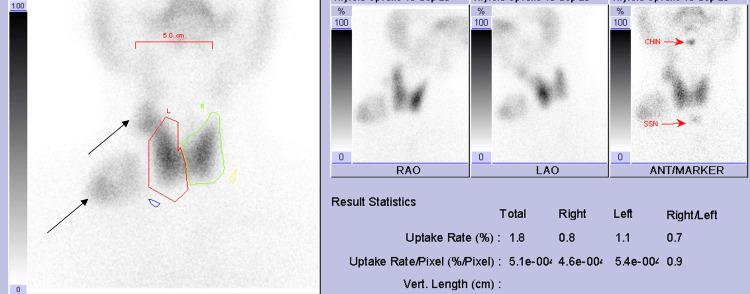
Planar images from thyroid uptake scintigraphy showing abnormal uptake in the right supraclavicular region and right mid-cervical region.

Thyroid uptake scintigraphy was performed, and it revealed heterogenous but normal thyroid global uptake (1.8%). Three right-sided neck soft tissue structures showing heterogenous uptake like the thyroid gland were also noted, the largest was in the supraclavicular region and is clearly separate from the right thyroid lobe (Figure 3).

**Figure 4:**
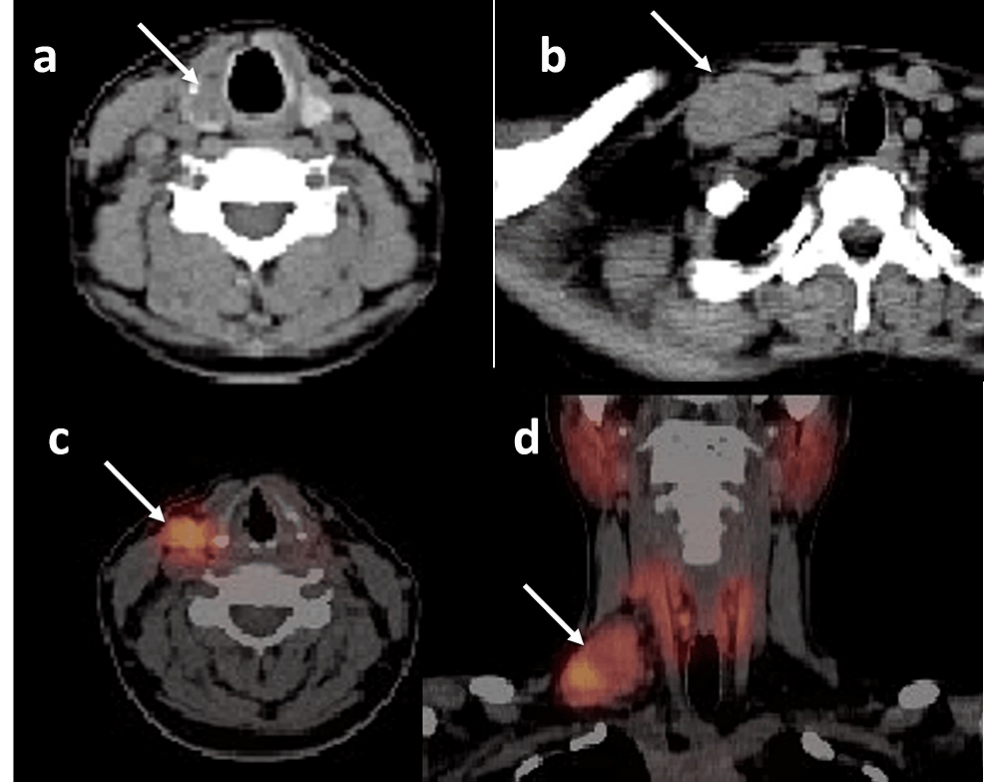
a. Axial CT images from the SPECT/CT study following thyroid uptake scan showing right thyroid lobe hypodense nodule (arrow). b: Axial CT images from the SPECT/CT showing right supraclavicular soft tissue lesion (arrow). c: Axial fused SPECT/CT image showing right level III lesion with uptake. d: Coronal SPECT/CT image showing uptake in the right supraclavicular lesion.

In SPECT/CT imaging (Figure 4), the areas of separate uptake on the right side of the neck were seen to correspond to soft tissue/nodal masses in levels III, IV, and supraclavicular regions. The largest one was in the right supraclavicular area and measured around 3.2x4.8 cm. The low-dose CT also revealed a right lobe hypodense thyroid nodule with focal calcification. The thyroid uptake findings confirmed the presence of ectopic thyroid tissue in the right cervical lesions. However, due to the suspicious ultrasound and SPECT/CT features, repeat ultrasound neck and FNAC were advised. The repeated ultrasound of the neck concluded that the supraclavicular lesion is likely to represent ectopic thyroid tissue due to similar echogenic characteristics with suspicion of developing diffuse thyroid disease within it (TR-2). The right thyroid lobe nodule is classified as TR-3. 

Conclusion and Results

In view of ultrasound and thyroid uptake findings and the inconclusive results of the fine needle aspiration, the patient was advised for an excisional biopsy of the right supraclavicular lesion to plan further management. Intraoperatively, there was a 2 cm hard nodule in the right thyroid lobe. The isthmus and left lobe appeared normal. There were no obvious lymph nodes in the central compartment. The frozen section histology from the right supraclavicular lymph node was reported as essentially normal thyroid tissue except for a small focus on papillary thyroid carcinoma.

**Figure 5:**
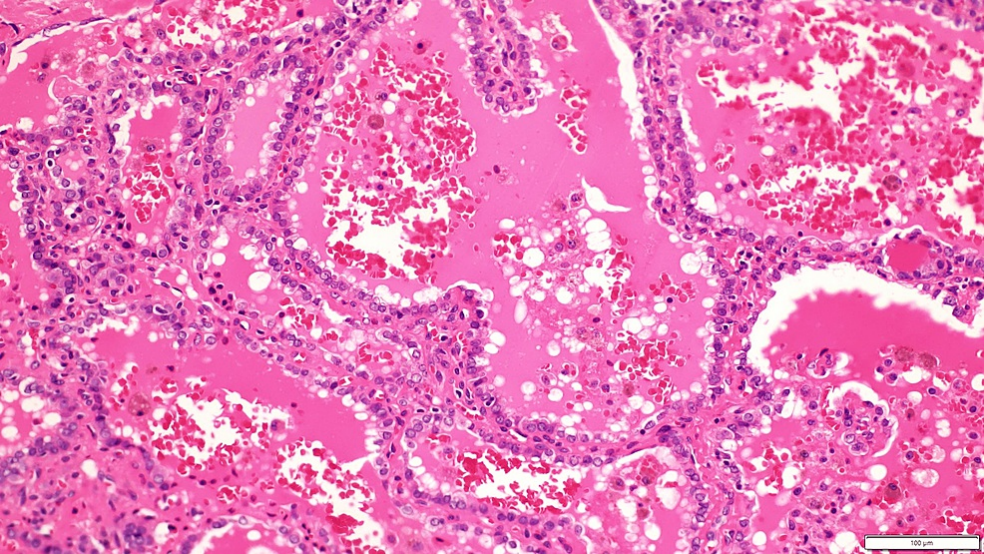
The image from the right lobe lesion shows nuclear features of papillary thyroid carcinoma, a classic variant arranged mainly in follicles.

**Figure 6:**
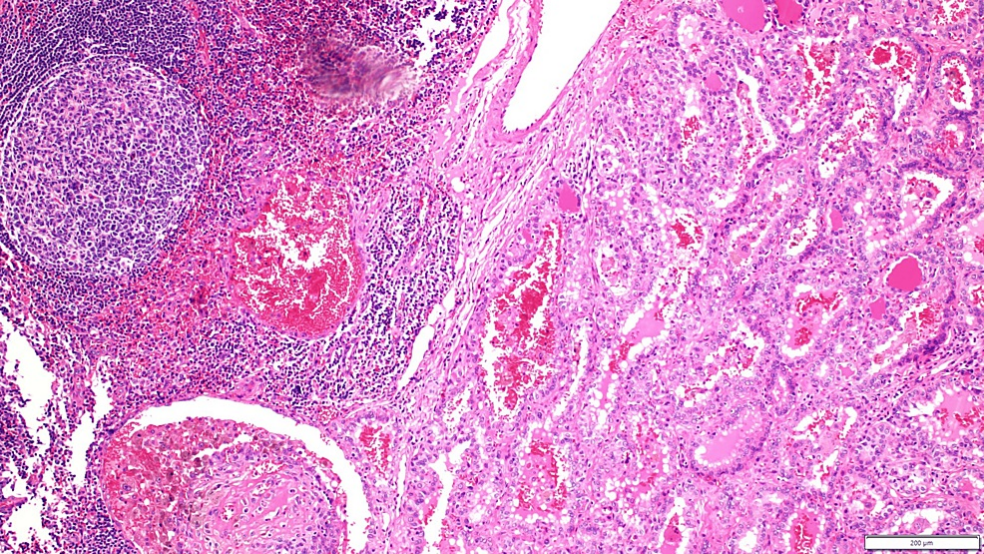
The picture shows a lymph node with a deposit of metastatic papillary thyroid carcinoma.

The decision at that time was to go for total thyroidectomy with right-side selective modified neck dissection. The final postoperative histopathology report concluded that the overall picture is of papillary thyroid carcinoma, a classic variant arranged mainly in follicles in the right lobe nodule (Figure 5), and metastatic papillary thyroid carcinoma in three out of 37 submitted right cervical lymph nodes (Figure 6). The pathological stage according to the 8th edition of AJCC (pTNM) is pT1bN1b. The patient was found to have papillary thyroid carcinoma with lymph node metastases.

Case 3

Case History/Examination

A 51-year-old man presented to primary health care with fever and neck pain. No other significant history was elicited, and the neck examination was unremarkable.

Methods

**Figure 7:**
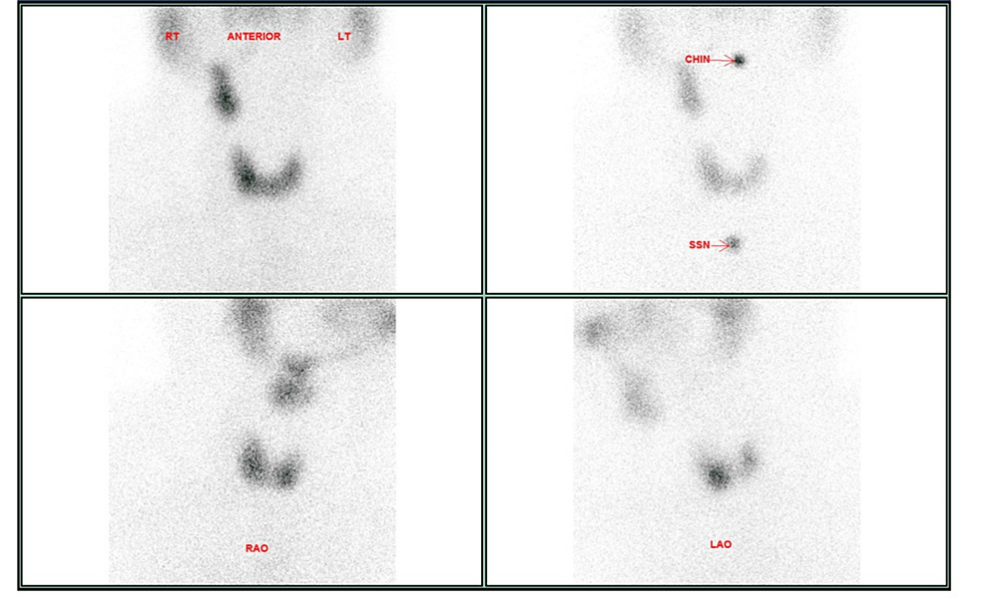
Anterior and bilateral oblique images of thyroid uptake scintigraphy showing a structure on the right side of the neck with similar uptake to the thyroid gland.

Investigations revealed elevated ESR levels. Thyroid function tests showed a suppressed TSH with a high thyroxin level. A thyroid ultrasound was performed and showed a few indeterminate hyperechoic nodules with rim calcifications and internal vascularity scattered in both lobes. However, there was no associated lymphadenopathy. He then underwent a thyroid uptake scintigraphy. Planar images of the neck showed a eutopic thyroid gland with diffuse heterogenous tracer uptake (normal global uptake) that could represent resolving thyroiditis or multinodular goiter with tiny nodules (Figure 7). 

**Figure 8:**
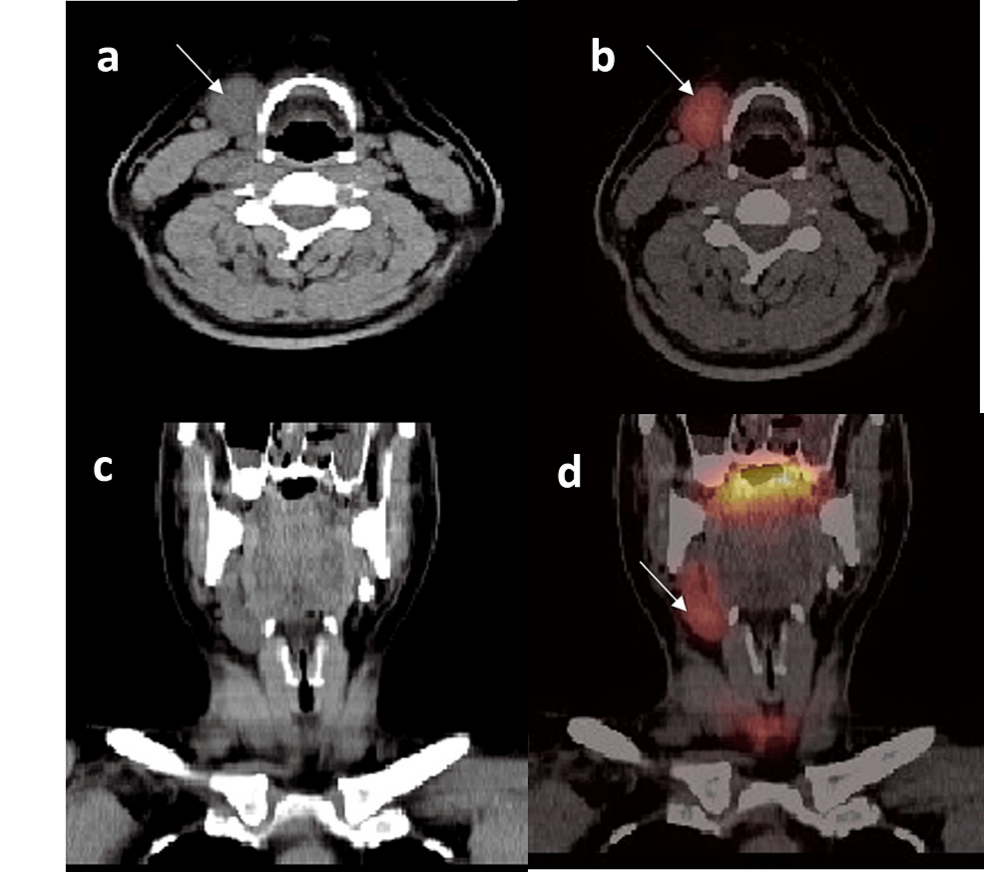
SPECT/CT images showing soft tissue structure (white arrow) on the right side of the neck with similar radiotracer uptake to the thyroid gland and absent submandibular gland on the left side. a) Axial unenhanced CT scan of the neck, b) axial fused SPECT/CT scan of the neck, c) coronal unenhanced CT scan of the neck, and d) coronal fused SPECT/CT scan of the neck.

An interesting incidental finding was also seen in the planar and confirmed in the SPECT/CT images. An elongated structure that showed a similar uptake to the thyroid gland was seen in the right upper cervical region. SPECT/CT imaging showed this uptake to correspond to an elongated hypodense soft tissue structure in right level III that does not have fatty hilum (Figure 8). No similar structures were seen in the neck.

The suspicion of ectopic thyroid tissue was speculated; however, the location of this structure and the absence of a submandibular gland on the left side raised the possibility of a hypertrophied right submandibular gland reaching down to the level III cervical region, which was confirmed on the SPECT/CT study.

*Conclusion and Results* This case was peculiar in terms of the uncertainty of the planar image findings that could have been very easily dismissed as possible ectopic thyroid tissue/gland. The SPECT/CT was very useful in that it did not only show the density and the exact relations of the structure but confirmed the absence of the left submandibular gland hence explaining the elongation/hypertrophy of the right submandibular gland. Due to the indeterminate nature of the thyroid nodules, fine needle aspiration of a right thyroid nodule was performed, and the results came as a benign follicular nodule (Thy2). The patient was continued on antithyroid medications.

## Discussion

The thyroid gland is normally anatomically located at the level of the second and fourth tracheal cartilages in the anterior neck region. Any presence of thyroid tissue in areas other than the normal location is ectopic. The prevalence of this is reported to be in 100000-300000 persons and occurs in 4000-8000 patients with thyroid disease. Sixty-five to eighty percent of cases occur in females. The coexistence with an eutopic thyroid gland is equal to that without one [1].

Only one study by Giuseppe Santangelo et al. documented 28 cases shown to have an ectopic thyroid gland, and 24 cases demonstrated concomitant eutopic thyroid glands [2]. There are no previous cases reported in our country; hence, we would like to highlight this correlation and share our characteristic imaging findings in these three cases with different outcomes. This in turn would raise radiologists' as well as clinicians’ awareness and certainty on this diagnosis, the investigations required, and the possible need for lifelong thyroid treatment.

The foramen cecum, at the posterior aspect of the tongue, is where the thyroid gland migrates to the infra-hyoid region during embryological development. This descent may come to a halt at any point in its course giving rise to an abnormally located (ectopic) thyroid gland. TC99m pertechnetate is normally distributed to the thyroid, gastric, salivary, and testicular tissues. Therefore, a soft tissue density mass along the migratory course of the thyroid gland showing uptake on TC99m pertechnetate thyroid scintigraphy is most likely an ectopic thyroid gland [2]. Usually, this is confirmed by doing fine needle aspiration but not all cases are subjected to such investigation. Ectopic thyroid tissue shows similar macro and microscopic features to a eutopic thyroid gland. Hence, they may be afflicted by the same pathological diseases or transformations [3]. Similar studies before on the coexistence of an ectopic and eutopic thyroid glands were published, one by Wei Zheng et al. and another by Khan et al. [1,4]. However, these were slightly different from our cases in the sense that in the former study, the ectopic tissue was found to be non-functional while the eutopic gland was normally functioning and in the latter study both thyroid glands were hyperfunctioning, contrary to our cases where the ectopic thyroid tissue was operating within the norm (uptake wise).

Eleni Kousta et al. published a case in Hormones back in 2005, where the ectopic thyroid gland was normal while the eutopic gland was found to function abnormally [3]. The patient was a young female who presented with a painless neck mass. Her ultrasound findings were suggestive of a normally located multinodular goiter. The limitations of these case report series are that TC99m pertechnetate is not specific to thyroid tissue, and we lack a definitive tissue biopsy sample in two cases for the reasons outlined in each case. Therefore, we recommend a tissue biopsy to be carried out for academic purposes in aid to confirm this rare entity and underline the possibility of it undergoing a disease process.

## Conclusions

The concurrent presence of both an ectopic and eutopic thyroid gland with abnormal activity of either is a unique phenomenon. This should be brought to the awareness of all endocrinologists, sonographers, and radiologists. We are highly in need of rich data and research on the etiologies and pathophysiology of such coexistence and possible disease transformation.
